# Time trends in hospital discharges in patients aged 85 years and older in Spain: data from the Spanish National Discharge Database (2000–2015)

**DOI:** 10.1186/s12877-021-02335-2

**Published:** 2021-06-16

**Authors:** Sergio Palacios-Fernandez, Mario Salcedo, Gregorio Gonzalez-Alcaide, Jose-Manuel Ramos-Rincon

**Affiliations:** 1 Department of Internal Medicine, San Juan de Alicante Hospital, Alicante, Spain; 2 Department of Internal Medicine, San Pedro Hospital, Logroño, Spain; 3 Department of History of Science and Documentation, University of Valencia, Valencia, Spain; 4 Department of Internal Medicine, General University Hospital of Alicante-ISABIAL, Alicante, Spain; 5 Department of Clinical Medicine, Miguel Hernandez University of Elche, Alicante, Spain

**Keywords:** Aged, 80 and over, Spain, Hospital mortality, Hospitalization, Elderly, Trends, Diagnoses

## Abstract

**Background:**

The aging population is an increasing concern in Western hospital systems. The aim of this study was to describe the main characteristics and hospitalization patterns in inpatients aged 85 years or more in Spain from 2000 to 2015.

**Methods:**

Retrospective observational study analyzing data from the minimum basic data set, an administrative registry recording each hospital discharge in Spain since 1997. We collected administrative, economic and clinical data for all discharges between 2000 and 2015 in patients aged 85 years and older, reporting results in three age groups and four time periods to assess differences and compare trends.

**Results:**

There were 4,387,326 discharges in very elderly patients in Spain from 2000 to 2015, representing 5.32% of total discharges in 2000–2003 and 10.42% in 2012–2015. The pace of growth was faster in older age groups, with an annual percentage increase of 6% in patients aged 85–89 years, 7.79% in those aged 90–94 years, and 8.06% in those aged 95 and older. The proportion of men also rose (37.30 to 39.70%, *p* < 0.001).

The proportion of patients that died during hospital admission decreased from 14.64% in 2000–2003 to 13.83% in 2012–2015 (*p* < 0.001), and mean length of stay from 9.98 days in 2000–2003 to 8.34 days in 2012–2015.

Some of the most frequent primary diagnoses became even more frequent relative to the total number of primary diagnoses, such as heart failure (7.84 to 10.62%), pneumonia (6.36 to 7.36%), other respiratory diseases (3.87 to 8.49%) or other alterations of urinary tract (3.08 to 5.20%). However, there was a relative decrease in the proportion of femoral neck fractures (8.07 to 6.77%), neoplasms (7.65 to 7.34%), ischemic encephalopathy (6.97 to 5.85%), COPD (4.23 to 3.15%), ischemic cardiomyopathy (4.20 to 8.49%) and cholelithiasis (3.07 to 3.28%).

**Conclusions:**

Discharges in the very elderly population are increasing in both relative and absolute terms in Spanish hospitals. Within this group, discharged patients are getting older and more frequently male. The mean length of stay and the proportion of patients that died during hospital admission are decreasing. Acute-on-chronic organ diseases, neoplasms, acute cardiovascular diseases, and infections are the most common causes of discharge.

**Supplementary Information:**

The online version contains supplementary material available at 10.1186/s12877-021-02335-2.

## Background

The elderly population, and especially those aged 85 and over (the very elderly), is the fastest-growing age segment in relative terms in Spain according to the National Statistics Institute (NSI) and the Human Mortality Database [1, 2]. In countries like Spain, where life expectancy exceeds 80 years of age, this trend is of the utmost importance. According to the NSI, people aged 85 years or older accounted for 3.2% of the total population in Spain in 2019 [1]. Their absolute number increased by 33% in recent years, from 2,258,317 in 2008 to 3,006,352 in 2018, while the number of people aged 95 or older increased by 80% in the same period [3]. In addition, elderly patients have the most healthcare requirements and incur the highest health expenditure, with about half the health expenditure in developed countries devoted to patients over the age of 65 [4].

In the hospital setting, trends show increasingly frequent discharges at all ages; this pattern is especially pronounced in the elderly population [5]. Moreover, hospital care for this population segment involves special challenges, including more frequent clinical complications during the hospital stay and patients who have cognitive and functional impairment. These challenges require tailored efforts from hospital staff, which sometimes have a limited benefit for the patient.

In this context, the World Health Organization (WHO) and the Spanish Ministry of Health have developed strategies for improving quality of life in older people through prevention and better management of major chronic diseases and disability [6]. These interventions have been applied in the last few years and could have had an impact on hospitalization trends in very elderly people.

As examples, the National Health System has adopted the Strategy for Health Promotion and Prevention, which aims to generalize public health measures, improve territorial coordination, empower populations, and create healthy and safe environments for children and the elderly. The strategy also addresses factors such as nutrition, physical activity, alcohol and tobacco consumption, and environmental safety [7]. Similarly, the National Strategy for Addressing Chronicity, last updated in 2012, lays out an intersectoral approach for stimulating health promotion, autonomy, and self care; decreasing the prevalence of risk factors for chronic diseases; and promptly diagnosing chronic conditions [8].

Collecting data on hospital discharges in elderly patients at the national level is relevant for understanding the magnitude and trends of hospitalizations in very elderly individuals. It will also elucidate some of the characteristics of these hospitalizations, such as gender distribution, trends according to age segment, and the main causes of hospitalizations. Finally, it will provide other information such as the proportion of patients that died during hospital admission, mean length of stay, and costs. To our knowledge, there are no epidemiological studies available addressing this issue in Spain. Describing hospital discharges, outcomes, and trends in elderly people could enable comparisons of health outcomes with other healthcare systems and inform planning for future provision of care.

In 2000, the NSI set average life expectancy in Spain at 75.9 years in men and 82.7 years in women [9]. It is of interest to know the main characteristics of those who outlived their life expectancy, so we chose to analyze discharges in patients aged 85 years or more, since this could reveal a specific pattern.

The aim of this study was to describe trends in the annual proportion of discharges, length of hospital stay, cost per hospital stay, in-hospital mortality and main diagnoses causing hospitalization in all patients aged 85 years or more who were hospitalized in Spain from 2000 to 2015.

## Methods

Since 1997, basic information has been collected for all patients discharged from Spanish hospitals, using an administrative system called the minimum basic data set (MBDS), which is managed by the Ministry of Health. The MBDS includes demographic data (age and sex), the date of admission and discharge, the primary diagnosis, up to 13 secondary diagnoses, the circumstances of discharge (general practitioner, voluntary discharge, home, death, etc.), up to 20 procedural codes, and an estimate of the cost per hospital stay using the diagnosis-related groups (DRG), a health economics concept used to delineate a set of diseases requiring analogous management resources. Until 2016, when the MBDS implemented the most recent codes from the International Classification of Diseases (ICD), 10th revision [10], all procedures and diagnoses were coded in using the (ICD-9-CM [11] so this is the version of the ICD we used.

We included all discharges in patients aged 85 years or older to any Spanish hospital between 2000 and 2015, which are described as the number of discharges. We performed an individual analysis of three age groups: 85–89 years, 90–94 years, and ≥ 95 years, although to calculate the proportion of patients that died during hospital admission, we made a group of 95–99 years instead of ≥95 years to prevent bias resulting in changes to the mean age of the group along the study period. The annual percentage of hospital discharges in very elderly people was considered the number of discharges in the study population per 100 adult discharges each year. Adults were considered all patients aged 15 years or more. Total discharges were considered the number of overall discharges in Spain each year.

We calculated the annual cumulative incidence by dividing the number of hospital discharges per year by the corresponding number of people in that population group in Spain at the beginning of the year according to the NSI annual report [1]. The cumulative incidence was expressed per 100 inhabitants. The proportion of patients that died during hospital admission was calculated as the number of discharges of patients aged 85 years or more who died during their hospital stay, divided by the number of annual discharges in patients aged 85 years or more. The mean length of hospital stay and costs per hospital stay were also estimated for each study year. Costs were calculated using diagnostic-related groups (DRG). All costs shown were adjusted for inflation during the same period in Spain to make them comparable with costs in 2000.

### Analysis of primary diagnoses

We conducted a temporal trends study of the most frequent primary diagnoses in very elderly hospitalized patients. We calculated the annual proportion (number of the specific diagnosis per 100 primary diagnoses in adults aged ≥85 years for every year) for each ICD-9 category in 2000, and we selected the 10 most frequent primary diagnoses in that year; these were used as a basis for the designation of 10 clinically related ICD-9 categories.

### Analysis of trends

To facilitate the analysis of trends, we grouped the data in four periods of 4 years. We also calculated the average annual percentage change (AAPC), with the following formula:

$$ \mathrm{AAPC}=\Sigma \mathrm{bi}/\mathrm{x}-1 $$

where bi is the year-to-year relative change in the annual proportion, risk estimation or cumulative incidence, as calculated using the following formula:

$$ \mathrm{bi}=\left(\mathrm{ni}-\mathrm{nh}\right)/\mathrm{nh} $$

where ni is the number of patients 1 year, the risk estimation for 1 year, or the cumulative incidence for that year; nh is the same magnitude from the previous year, and x is the number of years analyzed.

To better understand the evolution of hospitalization patterns in the ≥85 population (percentage of hospitalizations, proportion of patients that died during hospital admission, and mean length of stay), we made a linear graphic for each of these variables, shown as supplementary material.

Subsequently, we analyzed the time trends for the 10 selected disease categories. We compared the annual proportion for each diagnosis between 2000 and 2015 using the chi-squared test for trends.

Finally, to determine the most probable future trend to 2030, we tested the correlation coefficient for the annual proportion of each diagnostic group and the years of the study period. We considered an adequate correlation coefficient to be between 0.65 and 1 or − 0.65 and − 1. For results between 0.64 and − 0.64, we analyzed the cause of the lack of correlation by observing the time trend in a linear graphic, aiming to distinguish the stability of annual proportion over time from heterogeneous changes in trend. Finally, we formulated the best trend line for the known annual proportion and estimated the annual proportion up to 2030, using the tool included in Microsoft Excel Version 14.

### Statistical analysis

A descriptive statistical analysis of the most relevant characteristics of the studied population was performed. Quantitative variables were expressed as means, and qualitative variables as frequencies and percentages. Comparisons were performed using the chi-squared test or student t-test, as appropriate. We considered *p* values less than 0.05 to be statistically significant.

All the data were grouped in four periods of 4 years to facilitate their presentation. The numbers presented are the average values of the 4 years in each group.

### Ethical considerations

Data were treated with full confidentiality according to Spanish legislation. Patient identifiers were removed by the Spanish Ministry of Health before sharing the database with authors in order to strictly protect patient confidentiality. Given the anonymous and mandatory nature of the dataset, it was not necessary to obtain informed consent. The Spanish Ministry of Health evaluated our research protocols and determined that the anonymous database met all requirements according to Spanish legislation.

## Results

We identified 4,387,326 discharges of patients aged 85 years or more in Spain from 2000 to 2015, comprising 7.66% of total discharges. Two-thirds of those discharges were in patients aged 85–89 years; around 25%, 90–94 years; and 10%, 95 years or older.

Tables 1, 2, and 3 show the trends in the number of discharges, their distribution by age and sex, the results for the proportion of patients that died during hospital admission by age group and in relation with proportion of in-hospital deaths in all adults, cost analyses (corrected for inflation) by age group, and mean length of hospital stay.

**Table 1:** Hospitalization trends in people aged ≥85 years old in Spain, by five-year age bracket and four-year period, 2000 to 2015

Discharges in patients aged ≥85 years	2000–2003 (T1)	2004–2007 (T2)	2008–2011 (T3)	2012–2015 (T4)	AAPC
N (annual percentage of adult discharges^a^)	177,511 (5.32%)	227,630 (6.35%)	308,272 (8.29%)	383,417 (10.42%)	6.61%
*Discharges by sex, N (percentage of very elderly admissions)*					
Men	66,262 (37.34%)	85,298 (37.43%)	119,034 (38.59%)	152,291 (39.70%)	7.06%
Women	111,206 (62.63%)	142,316 (62.56%)	189,230 (61.40%)	231,120 (60.29%)	6.33%
*Discharges by age group, N (percentage of adult discharges)*					
85–89 years	120,034 (3.60%)	150,461 (4.19%)	207,176 (5.57%)	247,356 (6.72%)	6%
90–94 years	46,613 (1.39%)	61,964 (1.73%)	79,216 (2.13%)	109,331 (2.97%)	7.79%
≥ 95 years	10,864 (0.32%)	15,204 (0.42%)	21,879 (0.58%)	26,729 (0.73%)	8.06%
Cumulative incidence of discharges per 100 pop. Aged ≥85 years	25.54	28.94	31.19	31.41	1.91%
*Cumulative incidence by sex (admissions in men/women per 100 men/women in pop. Aged ≥ 85 years)*					
Men	31.59	36.01	38.46	38.48	1.8%
Women	22.91	25.89	27.89	28.01	1.89%
Population aged ≥85 years, N	694,442	784,708	988,153	1,219,485	4.61%

*AAPC* average annual percentage change

^a^The annual percentage of very elderly hospital discharges is calculated as: number of hospitalizations in patients aged 85 or more each year divided by the number of adult hospitalizations the same year

All variables were compared between adjacent time periods (T1 vs T2, T2 vs T3, and T3 vs T4), yielding *p* < 0.001 in all cases

**Table 2:** In-hospital mortality (N, %) in very elderly inpatients (≥ 85 years), by five-year age bracket and four-year period, 2000 to 2015

Deaths in very elderly inpatients	2000–2003 (T1)	2004–2007 (T2)	2008–2011 (T3)	2012–2015 (T4)	AAPC
N (annualized proportion of patients that died during hospital admission)	26,032 (14.63%)	33,837 (14.87%)	43,855 (14.23%)	53,059 (13.83%)	0.00%
85–89 years	15,914 (13.24%)	19,827 (13.19%)	26,003 (12.56%)	29,753 (12.03%)	−0.33%
90–94 years	7959 (17.02%)	10,702 (17.27%)	13,169 (16.64%)	17,654 (16.16%)	−0.03%
95–99 years	1926 (20.77%)	2955 (21.62%)	4172 (21.10%)	4952 (20.68%)	0.47%

*AAPC* average annual percentage change

All variables were compared between adjacent time periods (T1 vs T2, T2 vs T3, and T3 vs T4), yielding *p* < 0.001 in all cases

**Table 3:** Cost trends, corrected for the consumer price index (CPI), and length of hospital stay in very elderly inpatients, by five-year age bracket and four-year period, 2000 to 2015

	2000–2003 (T1)	2004–2007 (T2)	2008–2011 (T3)	2012–2015 (T4)	AAPC
Cost/discharge in inpatients aged ≥85 years (EUR)	4610.63	4941.58	5212.19	4824.12	1.2%
85–89 years	4587.54	4938.15	5236.08	4851.41	1.2%
90–94 years	4648.36	4941.43	5161.86	4776.99	1.07%
> 94 years	4708.27	4994.19	5178.68	4766.03	0.91%
% change, cost in inpatients aged ≥85 years relative to general adult inpatients	+ 13.88%	+ 8%	+ 2.23%	−0.44%	–
Total cost in inpatients aged ≥85 years/total cost in adult inpatients, %	6.06%	6.84%	8.45%	10.38%	4.67%
Mean length of hospital stay in patients aged ≥85 years, days (SD)	9.98 (11.06)	9.73 (10.40)	9.19 (9.69)	8.34 (8.40)	−1.27%
85–89 years (SD)	10.09 (11.20)	9.84 (10.73)	9.28 (9.26)	8.45 (8.55)	−1.26%
90–94 years (SD)	9.84 (10.78)	9.56 (9.76)	9.06 (9.64)	8.20 (8.21)	−1.26%
≥ 95 years (SD)	9.35 (10.14)	9.32 (9.72)	8.80 (9.26)	7.92 (7.89)	−1.22%
% change, mean length of stay in inpatients aged ≥85 years relative to general adult population	+ 24.95%	+ 25.42%	+ 23.90%	+ 18.87%	−0.35%
Mean difference in length of stay (inpatients aged ≥85 years – all adult inpatients), days	1.99	1.97	1.77	1.32	–

*AAPC* average annual percentage change, *CPI* consumer price index, *SD* standard deviation

All variables were compared between adjacent time periods (T1 vs T2, T2 vs T3, and T3 vs T4), yielding *p* < 0.001 in all cases

Between 2000 and 2015, the number of discharges in very old patients increased substantially, growing fastest the older the patient group (Table 1). Moreover, we observed an increase in the cumulative incidence of discharges per 100 population aged 85 years or more (supplementary Figure 1).

The proportion of patients that died during hospital admission in people aged 85 years or more decreased 0.8% along the study period. This declining trend began in T2 (Table 2 and supplementary Figure 2).

The economic analysis showed an increase in costs per hospital stay from 2000 to 2011 and a decrease in the final four-year period (Table 3). Costs were positively correlated with patient age. The ratio between costs in the very elderly and the general adult hospitalized population was positive in the first periods but then began to equalize.

Finally, the mean length of hospital stay decreased by 1.64 days from 2000 to 2003 to 2012–2015. Nevertheless, the average stay was positively correlated with patient age and was consistently longer in very elderly people compared to the general population of hospitalized adults (supplementary Figure 3).

### Time trends in the primary diagnoses

The most frequent ICD-9 diagnostic categories recorded at discharge were, in descending order: femoral neck fracture (ICD-9 820), heart failure (ICD-9 428), occlusion of cerebral arteries (ICD-9 434), pneumococcal pneumonia (ICD-9 481), chronic bronchitis (ICD-9 491), acute myocardial infarction (ICD-9 410), cholelithiasis (ICD-9 574), other disorders of the urethra and urinary tract (ICD-9 599), and other respiratory diseases (ICD-9 518).

In order to make the analysis clinically relevant, we created 10 groups by adding the annual proportions for related ICD-9 codes to the ones above. Moreover, we considered it likely that neoplasms were not included among the 10 most frequent primary disease categories due to the 99 categories into which this chapter is divided, so we added this significant chapter as a whole to the analysis. Table 4 describes the 10 most frequent clinically relevant groups, their annual proportion trend along the study period and the estimation of the annual proportion in 2030.

**Table 4:** Annual proportion trends (by 4-year period, 2000 to 2015) for the 10 most frequent primary ICD-9 diagnostic groups in inpatients aged ≥85, according to the MBDS, and projection for 2030

Disease (ICD-9)	Annual N (%) diagnoses in discharged patients aged ≥85				*P* value	AAPC	Correlation coefficient	Annual % of primary diagnoses in discharged patients aged ≥85 in 2030
	2000–2003	2004–2007	2008–2011	2012–2015				
Femoral neck fracture (820)	14,317 (8.07%)	17,320 (7.46%)	21,875 (7.10%)	25,911 (6.77%)	< 0.001	−2.10%	−0.97	3.19%
Heart failure (428)	13,925 (7.84%)	20,124 (8.63%)	30,031 (9.72%)	40,722 (10.62%)	< 0.001	1.34%	0.97	11.33%
Neoplasms (2)	13,581 (7.65%)	18,585 (7.99%)	24,639 (8.00%)	28,007 (7.34%)	< 0.001	−1.64%	− 0.81	5.07%
Ischemic encephalopathy (430–438)	12,367 (6.97%)	14,895 (6.41%)	19,543 (6.34%)	22,374 (5.85%)	< 0.001	−2.39%	−0.99	2.85%
Pneumonia (480–486)	11,303 (6.36%)	15,012 (6.46%)	20,940 (6.79%)	28,267 (7.36%)	< 0.001	0.67%	0.06	–
COPD (490–492)	7505 (4.23%)	8784 (3.80%)	10,217 (3.32%)	12,124 (3.17%)	< 0.001	−2.9%	−0.91	0.74%
Ischemic cardiomyopathy (410–414)	7473 (4.20%)	9377 (4.04%)	10,896 (3.54%)	12,028 (3.15%)	< 0.001	−2.65%	−0.93	0.9%
Other respiratory diseases (518–519)	6907 (3.87%)	14,532 (6.15%)	25,716 (8.34%)	32,556 (8.49%)	< 0.001	5.74%	0.86	12.99%
Other alterations of urethra and urinary tract (599)	5484 (3.08%)	8022 (3.43%)	14,050 (4.54%)	19,973 (5.2%)	< 0.001	3.64%	0,97	6.67%
Cholelithiasis (574)	5453 (3.07%)	7105 (3.06%)	10,137 (3.28%)	12,574 (3.28%)	< 0.001	−0.41%	−0.57	–

*AAPC* average annual percentage change (on percentage of total discharges), *COPD* chronic obstructive pulmonary disease, *ICD-9* International Classification of Diseases, 9th revision

**Fig. 1:**
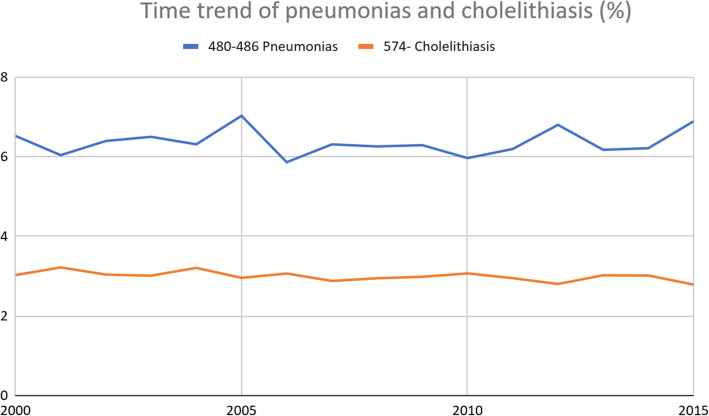
Time trend of pneumonias and cholelithiasis (%)

The correlation coefficient between date and annual proportion for each diagnostic group was adequate in all groups but pneumonia and cholelithiasis. We made a linear graphic with the known annual proportion of hospitalizations due to pneumonia and cholelithiasis between 2000 and 2015 (Fig. 1), which shows stability throughout the study period, with an AAPC of only 0.67 and − 0.41% respectively; thus, we considered pneumonia and cholelithiasis to show a stable trend during the study period, which will probably hold in the years to come.

As seen in Table 4, there was an increase in the annual proportion of heart failure (AAPC 1.34%), other respiratory diseases (AAPC 5.74%), and other disorders of the urethra and urinary tract (AAPC 3.64%). There was also a decrease in the number of discharges associated with femoral neck fractures (AAPC − 2.10%), neoplasms (AAPC − 1.64%), ischemic encephalopathy (AAPC − 2.39%), chronic obstructive pulmonary disease (COPD) (AAPC − 2.90%), and ischemic cardiomyopathy (AAPC − 2.65%). Cholelithiasis and pneumonia showed the same annual proportion during the study period. The correlation coefficients and the annual proportion of each diagnosis are presented in Table 4. According to current trends, the 10 primary diagnostic groups will comprise around 53% of total diagnoses at discharge in 2030.

We also analyzed the 10 most frequent primary diagnoses in 2015, observing that ischemic cardiomyopathy fell to the 11th position. On the other hand, heart arrhythmias ranked 7th in 2015, compared to 12th in 2000.

## Discussion

This study shows some important features about very elderly hospitalized patients in Spain: (i) there has been a significant absolute and relative increase in hospitalizations in patients aged 85 or more; (ii) hospitalizations are more frequent in women than men, but the gap is closing progressively; (iii) the proportion of patients that died during hospital admission is decreasing in the very old population, as is the length of stay; (iv) neoplasms, hip fractures and the respiratory and circulatory diseases are the primary diagnoses causing hospitalization.

Previous studies analyzing trends in hospitalized patients have focused on specific diseases [12, 13] or single centers, limiting the size of the population analyzed [14, 15]. To our knowledge, this is the first study to analyze the general trends in very elderly hospitalized patients in Spain, although the Spanish Ministry of Health does periodically provide a summary of the information contained in the MBDS. In 2012, they analyzed the characteristics of hospitalizations in patients aged over 65; as in our study, their results showed an increase in that population group, combined with a decrease in the mean length of stay, comparable to what we observed in the very elderly population [16]. We also found that according to the AAPC, the number of discharges in very old patients is increasing fastest in the oldest age segments, with the most rapid increase in people aged 95 and older.

Our study shows a progressive increase in the cumulative incidence of hospitalizations among the population aged 85 or more. Since these results are not age adjusted, it is possible that the increase is explained by the progressive expansion of the oldest age groups. This observation demonstrates that this age group is not only increasing in number but also in healthcare requirements.

There is a wide gap between the number of men and women in the analyzed population, with men accounting for just over a third of the very old inpatients. The reasons for this imbalance probably reside in the differential life expectancy between sexes and the subsequent smaller proportion of men among very elderly inhabitants in Spain, even though men had a higher cumulative incidence of discharges than women. This result is consistent with other recent studies that show differences in hospitalization patterns depending on sex in elderly patients: women discharged in Spanish hospitals tend to be older and with lower Charlson index scores than men [17]. The summary of the MBDS by the Ministry of Health showed that in 2012, the proportion of hospitalizations in men aged 65 to 84 is higher (51% in 2010) than among those aged 85 or more (35% in the period 2008–2011) [16].

Our study showed a progressive decrease in the mean length of stay among very old inpatients, which is consistent with the summary results provided by the Ministry of Health in 2012 [16]. Similarly to our study, the US Agency for Healthcare Research and Quality (AHRQ) data show that very elderly people made up 8% of inpatients in 2008, although they showed a lower mean length of stay (5.6 days in the USA versus 9.19 days in Spain) [18]. In 2017, Brandão et al. published a study analyzing the administrative data of patients aged 80 years or older and hospitalized between 2000 and 2014 in public acute care Portuguese hospitals [19]. Authors observed a lower length of stay among their nonagenarian hospitalized population than the Spanish nonagenarian group (7 versus 9.5 days). The variations observed with respect to our results could be due to differences between healthcare systems, but they could also signal an opportunity to improve Spanish hospital care.

With regard to the proportion of patients that died during hospital admission, this has been estimated at 8.2% in those older than 65 years [15], compared to 13.3% in patients older than 90 [20]. In 2015, Pires-Machado et al. observed an in-hospital mortality rate of 19.7% among inpatients aged 80–89 in Brazil, substantially higher than the 12.6% in the 85–89-year age bracket in our study [21]. An Israeli study published in 2010 on hospitalized nonagenarians reported an in-hospital mortality rate of 28%, which is higher than our results [22]. In 2013, Ramos et al. analyzed the mortality of nonagenarians discharged from the internal medicine ward of a Spanish tertiary hospital, finding a crude mortality rate of 27.7% [23]. Other studies focus their analysis on specific causes of hospitalization. For example, in 2012 Gur et al. analyzed the in-hospital outcomes of patients aged 85 years or older after a first-ever ischemic stroke and found an in-hospital mortality rate of 11.3% over the study period, with a dramatic decrease from 2004 (18.7%) to 2010 (5.7%). Previously published data on in-hospital mortality among elderly patients is difficult to compare with our data due to economic differences between the studied countries and methodological heterogeneity, for instance the use of crude estimations for mortality or data regarding a particular diagnosis [24].

According to the MBDS data, the proportion of patients that died during hospital admission among very old inpatients started declining around the 2003–2007 period. However, due to the increase in the very old hospitalized population, the proportion of deaths in inpatients aged 85 years and older has progressively increased in relation to total adult in-hospital deaths. It is possible that the increase in the life expectancy in Spain (from 75.9 years in 2000 to 79.9 in 2015 for men and from 82.7 years in 2000 to 85.4 in 2015 for women) explains the decrease in the proportion of deaths and the increase in the share of total in-hospital deaths of the study population [25].

Regarding the causes of hospitalization, there is some overlap between our data and the summary from the Spanish Ministry of Health: diseases of the circulatory and respiratory systems; neoplasms; and diseases of the muscular, skeletal and genitourinary apparatus were among the most frequent primary diagnoses. However, wounds and poisoning was the fifth most frequent chapter in the Ministry report, while this category did not even rank among the top 10 primary diagnoses at discharge in our study. This difference could be explained because people tend to cut down on their activity levels as they age, so very elderly people carry a lower risk of wounds or poisoning. Other differences reside in the chapters related to symptoms, signs and poorly defined states as well as endocrine, nutritional, and metabolic diseases, which were among the most frequent diagnoses in the Ministry summary but were much less prominent in our study population.

In 2008, the AHRQ published a statistical brief that presented data from the Healthcare Cost and Utilization Project [16]. Authors described patient characteristics and hospital utilization among the oldest adults, including those aged 85 years and older. Diagnoses were quite similar to those in our cohort, with heart failure, pneumonia, urinary tract infection, hip fracture, stroke and COPD among the 10 most frequent diagnoses. However, blood infection (septicemia), kidney failure, and electrolyte and water misbalances were also top diagnoses in the USA, unlike in Spain. Variations in coding practices between countries could explain some of this difference.

Finally, in 2017 Brandão et al. observed that among Portuguese nonagenarians the 10 most frequent primary diagnoses were, in descending order of frequency, pneumonia (17.7%), femoral neck fracture (7,7%), acute cerebrovascular disease (6.8%), heart failure (6%), urinary tract infections (5.2%), acute bronchitis (3.6%), other lower respiratory disease (3%), biliary tract disease (2.3%), fluid and electrolyte disorders (2.2%) and chronic obstructive pulmonary disease and bronchiectasis (1.9%) [26]. These primary diagnoses are similar to the ones found in our study, with the exception of fluid and electrolyte disorders.

The MBDS data show an increase in all causes of discharge, since the number of discharges went up. However, there is a downward trend in the annual proportion of some of the most frequent diagnoses in Spanish hospitals, including femoral neck fractures, COPD, ischemic encephalopathy, and ischemic cardiomyopathy. This decline may be attributed to different causes, such as the effectiveness of public health policies, which have addressed primary and secondary prevention for diseases related to vitamin D deficiency, osteoporosis, hypertension, diabetes, obesity, tobacco and alcohol consumption, diet, insufficient physical activity and dyslipidemia [7, 8]. Other possible reasons include improvements in screening for conditions that increase the risk of these diseases, or cohort or period effects (that is, that very elderly Spaniards are generally healthier as time goes by). Some studies have found similar results when focusing on the evolution of some of these diseases. For example, Lewieck et at. observed a decrease in the incidence of hip fractures in the USA from 2002 to 2015 [27]. In 2015, Moran et al. published a manuscript evaluating the incidence of myocardial infarction and angina, which showed a general decrease around the world [28]. There have been similar findings in the case of ischemic encephalopathy [29] and COPD [30].

Hospitalization costs changed significantly during the study period. The pronounced increase from 2000 to 2007 was attenuated in 2008–2011 before dropping in 2012–2015. This reversal could be due to the health budget adjustment in Spain following the 2008 economic crisis. Mean length of stay gradually decreased between 2000 and 2015 in all age segments, coinciding with efforts from within the Spanish healthcare system to improve the efficiency of hospital care. The analysis of the AHRQ shows significantly higher costs per hospitalization in the USA with respect to our data (USD 9400 versus EUR 5212, respectively), probably related to higher healthcare costs and the dominance of private providers in the USA.

The strengths of our study lie in the large sample size, the 15-year follow-up period, and the reliability of the data, which come from a well-established administrative system. However, some limitations should be considered when interpreting its results. The source of our data is the MBDS, an administrative database that includes data collected from each discharge report in Spain. This kind of database is of good quality for administrative data, but it shows low sensitivity and high specificity for clinical data such as primary diagnoses [31, 32]. Since this study only analyzes the primary diagnoses, which are compulsory in every discharge report, it is unlikely to be affected by the lack of sensitivity; however, it is possible that some diagnoses at discharge are not accurate or are included in non-specific items in the ICD-9, which could lead to an overconcentration of diagnoses.

Another limitation is the anonymity of the database (patients are not identified by clinical history number or name), which prevents any analysis of readmissions; moreover, patients who were transferred from one hospital to another would have duplicate entries. Since we cannot differentiate new admissions from recurrent admissions, it is difficult to draw conclusions regarding the cause of the changes in the frequency of hospitalizations.

In the clinical field, the database is limited because it uses ICD-9-CM diagnostic codes to identify the primary cause of hospitalization. The major concern in this case is the questionable accuracy of these diagnoses, which cannot be verified.

Despite these limitations, the MBDS discharge data is a mandatory register with an estimated coverage of 98% [26]. Data are also audited periodically, minimizing inaccuracies. Finally, the Spanish health system provides universal healthcare coverage, allowing the standardization of data from patients with different socioeconomic backgrounds or living in different Spanish regions.

## Conclusions

Hospital discharges of very elderly patients in Spanish hospitals are rapidly increasing, and the growth rate is higher among the oldest patients. The classic gap in the proportion of hospitalizations among very old women and men is progressively closing. The proportion of patients that died during hospital admission and the average stay are decreasing. Finally, the most prevalent diagnoses causing hospitalizations in this age group are neoplasms, heart failure, ischemic cardiomyopathy, pneumonia, COPD, other respiratory diseases, femoral neck fracture, other alterations of the urethra and urinary tract, and cholelithiasis.

## Supplementary Information

**Additional file 1: Supplementary Figure 1.** Hospitalization annual trends in people > 85 years old in Spain per 100 global discharges by age group, from 2000 to 2015. **Supplementary Figure 2.** Annual trends in people > 85 years of the proportion of deaths per age group, from 2000 to 2015. **Supplementary Figure 3.** Annual trends in people > 85 years of mean length of stay per age group, from 2000 to 2015

## Data Availability

The data that support the findings of this study are available from the Spanish Ministry of Health, but restrictions on the availability of these data apply. In our study they were used under license and so are not publicly available. Ramos-Rincon J.M. has full access to and is the guarantor for the data. The datasets generated are available from the corresponding author on reasonable request and with permission of the Spanish Ministry of Health. The summarized data that support the findings of this study are publicly available from [Spanish Ministry of Health: Hospital Discharge Records in the National Health System. CMBD: https://www.mscbs.gob.es/en/estadEstudios/estadisticas/cmbdhome.htm].

## Abbreviations

NSI: National Statistics Institute
MBDS: Minimum basic data set
WHO: World Health Organization
ICD-9: International Classification of Diseases, 9th revision
AAPC: Annual Average Percentage Change
COPD: Chronic obstructive pulmonary disease
AHRQ: Healthcare Research and Quality
DRG: Diagnosis-Related Groups
