# Supplementary material for: Time trends in hospital discharges in patients aged 85 years and older in Spain: data from the Spanish National Discharge Database (2000–2015)
Source: BMC Geriatr. 2021 Jun 16;21:371. doi: 10.1186/s12877-021-02335-2 (PMC8207637; doi:10.1186/s12877-021-02335-2)

**SUPPLEMENTARY FIGURE 1**. Hospitalization annual trends in people > 85 years old in Spain per 100 global discharges by age group, from 2000 to 2015


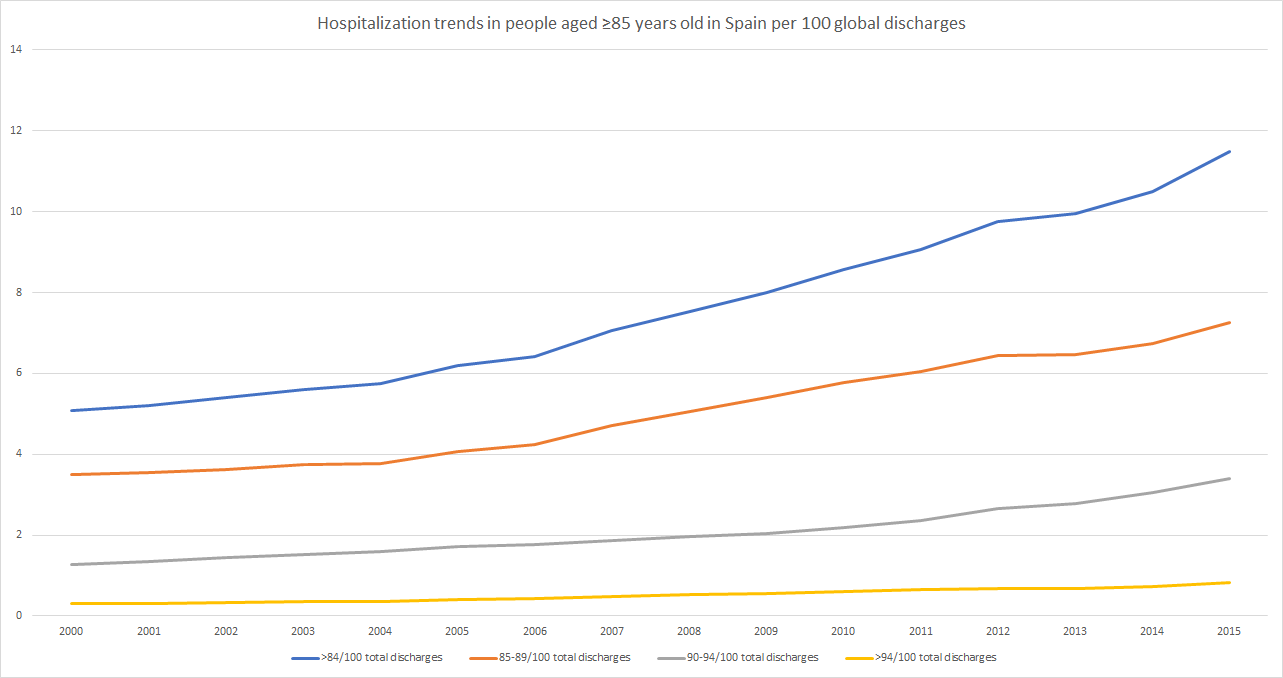


**SUPPLEMENTARY FIGURE 2**. Annual trends in people > 85 years of the proportion of deaths per age group, from 2000 to 2015


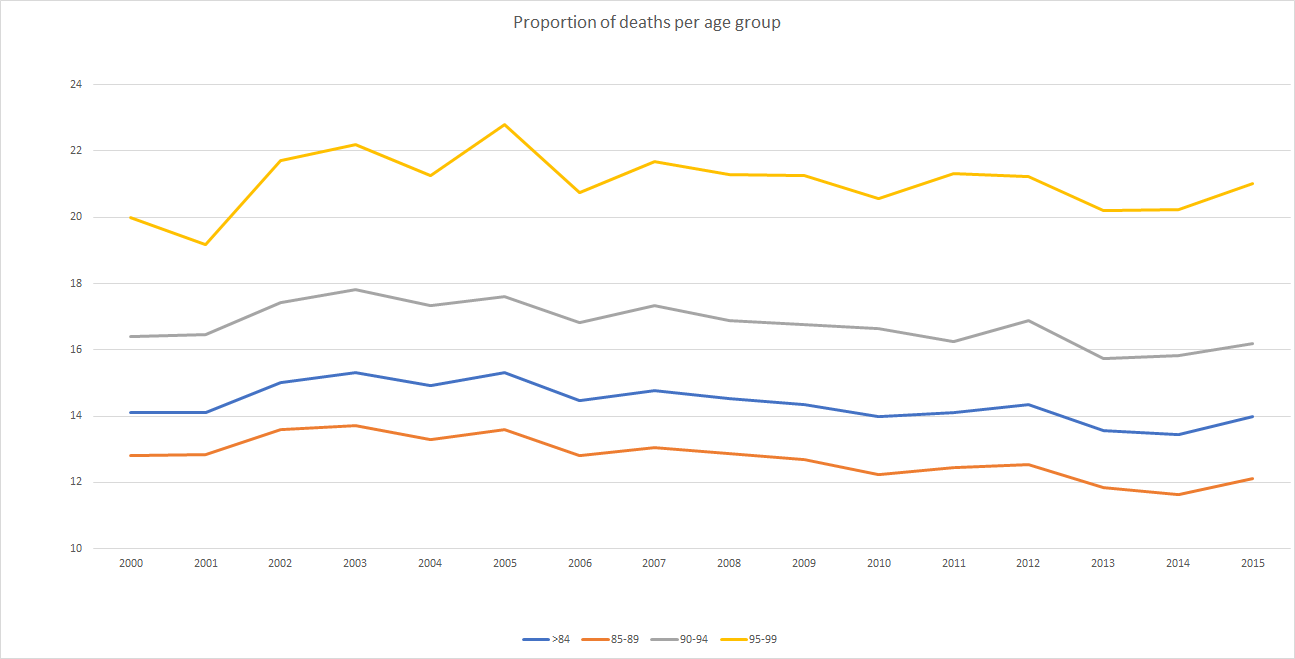


**SUPPLEMENTARY FIGURE 3**. Annual trends in people > 85 years of mean length of stay per age group, from 2000 to 2015


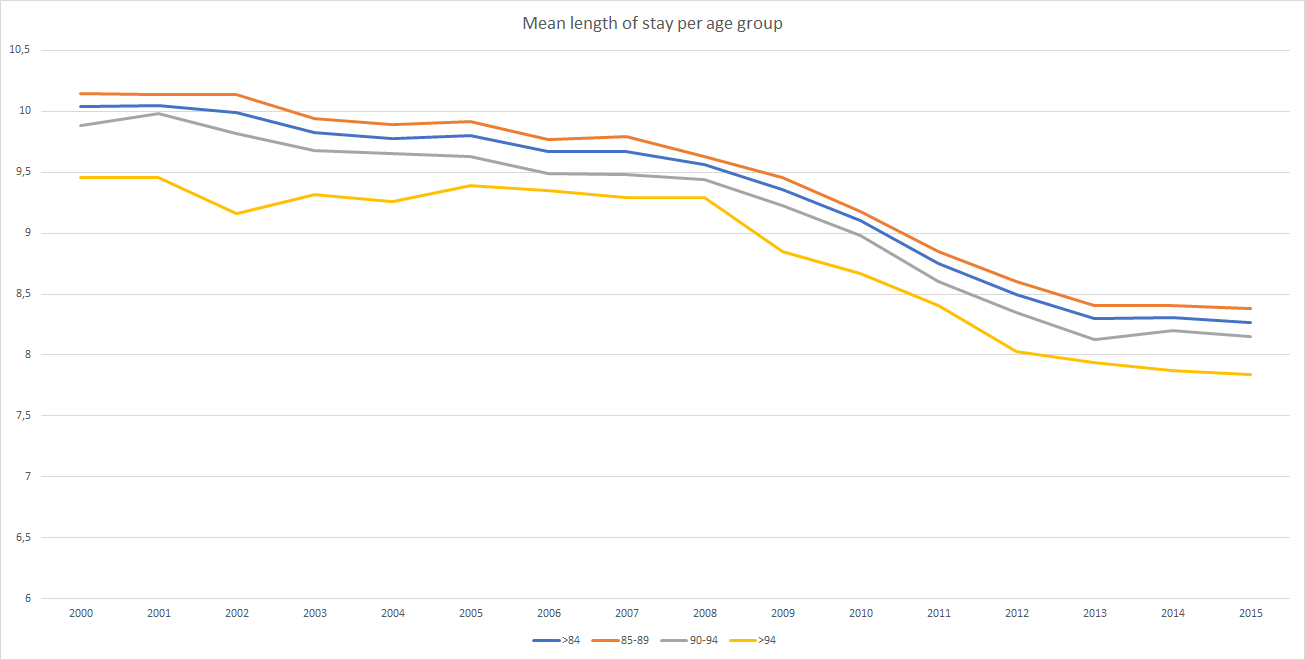

Supplement: Supplementary file 1 — Additional file 1: Supplementary Figure 1. Hospitalization annual trends in people > 85 years old in Spain per 100 global discharges by age group, from 2000 to 2015. Supplementary Figure 2. Annual trends in people > 85 years of the proportion of deaths per age group, from 2000 to 2015. Supplementary Figure 3. Annual trends in people > 85 years of mean length of stay per age group, from 2000 to 2015 [file 12877_2021_2335_MOESM1_ESM.docx]
